# Phase-Field Simulations at the Atomic Scale in Comparison to Molecular Dynamics

**DOI:** 10.1155/2013/564272

**Published:** 2013-12-19

**Authors:** Marco Berghoff, Michael Selzer, Britta Nestler

**Affiliations:** Institute of Applied Materials, Karlsruhe Institute of Technology, 76133 Karlsruhe, Germany

## Abstract

Early solidification is investigated using two different simulation techniques: the molecular dynamics (MD) and the phase-field (PF) methods. While the first describes the evolution of a system on the basis of motion equations of particles, the second grounds on the evolution of continuous local order parameter field. The aim of this study is to probe the ability of the mesoscopic phase-field method to make predictions of growth velocity at the nanoscopic length scale. For this purpose the isothermal growth of a spherical crystalline cluster embedded in a melt is considered. The system in study is Ni modeled with the embedded atom method (EAM). The bulk and interfacial properties required in the PF method are obtained from MD simulations. Also the initial configuration obtained from MD data is used in the PF as input. Results for the evolution of the cluster volume at high and moderate undercooling are presented.

## 1. Introduction

The phase-field is a powerful method to describe solidification phenomena [1, 2] on the mesoscopic length scale. It has been used to model homogeneous and heterogeneous nucleation, microstructure formation in solids, and motion of grain boundaries. The phase-field models includes formulations for pure substances [3], for multicomponent systems [4], and for polycrystalline structure [5] and solidification in eutectic [6, 7], peritectic [7], and monotectic [8] systems. Other simulation techniques for dendrite growth are cellular automata [9] and hybrid methods such as the multiscale diffusion Monte Carlo (DMC) [10, 11].

The phase-field method requires previous knowledge of the material properties of the system in study. The input includes bulk properties such as density, heat capacity and latent heat, and others such as interfacial and kinetic growth coefficients, being the latter properties which are hardly accessible in experiments. Here molecular simulations play a fundamental role, since they provide a link between an interaction potential and all the required properties. Kinetic coefficients, interfacial free energies, and their dependence on the crystal orientation, of pure metals and alloys, can be directly obtained from simulations of inhomogeneous liquid-crystal systems [12–15].

There are previous phase-field studies in which the material parameters have been obtained from molecular simulations to model dendritic growth in pure Ni [16, 17], CO_2_ hydrates [18], and binary alloys. In this work we compare MD and PF simulation results for the isothermal growth velocity of a nanoscopic spherical crystalline cluster of Ni embedded in the melt. For this purpose we use precise thermodynamic and kinetic data obtained from molecular dynamics and use equivalent initial configuration in the phase-field simulations.

## 2. Properties of Ni (EAM)

The properties of Ni obtained from molecular dynamic simulations are given in this section. The embedded atom potential of Foiles [19] is used. The thermal dependence of bulk properties is determined from independent simulations of equilibrated homogeneous systems at pressure *p* = 0. Liquid phase is prepared by melting of an initial crystalline phase, by setting the temperature of the thermostat well above the melting temperature. Crystalline phase is obtained by relaxation of the perfect crystalline phase. The samples are prepared in NpT simulation runs and the production in NVE ensemble.

Densities of the bulk phases are fitted to the function *ρ* ^*α*^(*T*) = *a* + *b* · *T* + *c* · *T* ^2^, for the fcc solid phase (*α* = s) *a* = 8901.6 kg/m^3^, *b* = −0.20379 kg/m^3^ K, and *c* = −0.0000614202 kg/m^3^ K^2^ while for the liquid phase (*α* = *ℓ*) *a* = 8992.26 kg/m^3^, *b* = −0.667037 kg/m^3^ K, and *c* = 0.0000331612 kg/m^3^ K^2^. The parameters were obtained in the temperature intervals 1000 K to 3000 K for the liquid and 300 K to 1900 K for the solid. In both cases parts of the metastable phases are included in the fit procedure.

The latent heat is obtained from the difference between the enthalpy of the liquid and the solid and fitted to ${\tilde{L}}_{0}(T)=a+b\cdot T+c\cdot T^{2}$ with *a* = −15980.2 J/kg, *b* = 324.745 J/kg K, and *c* = −0.0810984 J/kg K^2^. These values are determined in the range from 1000 K to 1900 K. The latent heat expressed in units of energy per volume of the solid phase is given by $L^{\text{s}}(T)={\tilde{L}}_{0}(T)\cdot \rho^{\text{s}}(T)$.

The specific heat capacity at constant volume is given by ${\tilde{c}}_{v}^{\alpha }(T)=a+b\cdot T$ where *a* = 419.452 J/kg K and *b* = 0.020388 J/kg K^2^ for the solid and *a* = 563.024 J/kg K and *b* = −0.06952 J/kg K^2^ for the liquid.

The thermal diffusivity for the model is 2.1 · 10^−7^ m^2^/s [20] while the experimental values range from 120 · 10^−7^ m^2^/s [21] to the recent value 8.7 · 10^−7^ m^2^/s [22] (obtained in microgravity conditions). The value obtained from simulations is at least 4 times lower than that in experiments, and the difference is due to the electronic contribution to thermal transport in metals which are not accounted in classical molecular dynamics.

The melting temperature of the model is precisely estimated from simulations of inhomogeneous solid-liquid system at the point where the velocity of growth is zero. For the model *T*_*m*_ = 1748 K.

The kinetic coefficient is estimated from linear relationship between planar growth velocity and undercooling close to coexistence. Growth velocities are obtained from independent simulations for the crystal orientations [100], [110], and [111]. The kinetic growth coefficient depends also on the orientation of the crystal. Its orientational dependence is represented here by the expansion

(1) $$\begin{array}{c} \frac{k\left(\hat{n}\right)}{k_{0}}=1-3\epsilon_{k}+4\epsilon_{k}Q+\delta_{k}\left(P+30S\right) \end{array}$$

with *Q* = *n*_1_^4^ + *n*_2_^4^ + *n*_3_^4^, *P* = *n*_1_^6^ + *n*_2_^6^ + *n*_3_^6^, and *S* = *n*_1_^2^ *n*_2_^2^ *n*_3_^2^. $\hat{n}=(n_{1},n_{2},n_{3})$ is the unit vector normal to the interface. The parameter *k*_0_ is a value of the magnitude of the kinetic coefficient; *ϵ*_*k*_ and *δ*_*k*_ are the strength of the anisotropy of the kinetic coefficient. The values for the model EAM F85 are *k*_0_ = 0.319205 m/s K, *ϵ*_*k*_ = −0.196511, and *δ*_*k*_ = 0.230331, estimated from the kinetic coefficients for different crystal orientations; *k*_100_ = 0.33 m/s K, *k*_110_ = 0.23 m/s K, and *k*_111_ = 0.12 m/s K.

The interfacial stiffness and interfacial free energy were obtained from the analysis of capillary waves spectrum of large solid-liquid interfaces at coexistence (i.e., at *T* = *T*_*m*_ for *P* = 0). The orientational dependence of the interfacial free energy *γ* is described by a cubic harmonic expansion

(2) γ(n^)γ0=1+ϵ1(Q−35)+ϵ2(3Q+66S−177) +ϵ3(5Q2−16S−9413Q+3313)

with *γ*_0_ being an orientational averaged value of the interfacial free energy, while the coefficients *ϵ*_*i*_ describe the strength of the anisotropy. Interfacial stiffness is defined as ${\tilde{\gamma }}_{\alpha \beta }=\gamma +\partial^{2}\gamma /\partial {\hat{n}}_{\alpha }\partial {\hat{n}}_{\beta }$, where *n*_*α*_ and *n*_*β*_ indicate unit vectors tangent to the interface plane [23]. The parameters of (2) are obtained from the system of equations for the stiffnesses ${\tilde{\gamma }}_{\alpha \beta }$ and their numerical values are obtained from the analysis of the capillary waves spectrum [14]. Here we consider the crystal orientations [100], $[{110]}_{\left[1\overset{-}{1}0\right]}$, [110]_[001]_, and [111]; the interface energy for the [110] orientation depends on the parallel direction denoted by the subscripts. In respect to the four different interface energies we used a cubic harmonic expansion with four fitting parameters. Accurate values of these variables and parameters for the model EAM F85 are *γ*_0_ = 0.302 J/m^2^, *ϵ*_1_ = 0.10191, *ϵ*_2_ = −0.00134, and *ϵ*_3_ = 0.00876.

## 3. Phase-Field Model for Pure Material

Isothermal crystal growth for pure material is modeled by the variables of the internal energy density *e* and of two phase-fields 0 ≤ *ϕ*_s_ ≤ 1 (solid) and 0 ≤ *ϕ*_*ℓ*_ ≤ 1 (liquid). The phase-field variables fulfill the constraint *ϕ*_s_ + *ϕ*_*ℓ*_ = 1, so a single phase-field variable *ϕ* = *ϕ*_s_ is sufficient to describe the evolution of the phase boundaries in the system. Note that *ϕ*_*ℓ*_ = 1 − *ϕ*. The variable $\phi (\vec{x},t)$ denotes the local fraction at $\vec{x}$ of the considered phase at time *t*.

The phase-field model is based on an entropy functional to ensure consistency with classical irreversible thermodynamics

(3) $$\begin{array}{c} 𝒮\left(e,\phi \right)=\int_{\Omega }s\left(e,\phi \right)-\left(\epsilon a\left(\phi ,\nabla \phi \right)+\frac{1}{\epsilon }w\left(\phi \right)\right)dx. \end{array}$$

The bulk entropy density *s* depends on the internal energy density *e* and the phase-field variable $\phi (\vec{x},t)$. The functions *a*(*ϕ*, ∇*ϕ*) and *w*(*ϕ*) reflect the thermodynamics of the interfaces and *ϵ* = 4 is a small length scale parameter related to the thickness of the diffuse interface.

In detail the gradient entropy with anisotropy is adopted from (2) and modeled by the factor ${\left(A\left(\hat{n}\right)\right)}^{2}$ depending on the orientation of the interface. Consider the following:

(4) $$\begin{array}{c} a\left(\phi ,\nabla \phi \right)=\frac{\gamma_{0}}{T}{\left(A\left(\hat{n}\right)\right)}^{2}{\left|\nabla \phi \right|}^{2}=\gamma \left(\hat{n}\right){\left|\nabla \phi \right|}^{2}, \end{array}$$

where $\hat{n}=-\nabla \phi /\left|\nabla \phi \right|$ is the normalized gradient vector.

The function *ω*(*ϕ*) = (16/*π* ^2^)(*γ*_0_/*T*)*ϕ*(1 − *ϕ*) is the obstacle potential. It is set that *ω*(*ϕ*) = *∞* for *ϕ* ∉ [0,1]. Note that *γ*_0_/*T* is the interface entropy density.

From (3) one derives the equations for the nonconserved phase-field variable *ϕ* by taking the functional derivatives in the form

(5) $$\begin{array}{c} \tau \epsilon \frac{\partial \phi }{\partial t}=\frac{\delta 𝒮}{\delta \phi }, \end{array}$$

where *τ* = *τ*(*ϕ*, ∇*ϕ*) is an anisotropic relaxation parameter dependant on temperature, $\tau (\hat{n})=L(T)/TT_{m}k\left(\hat{n}\right)$ with (1), and it is related to the kinetic coefficient *k*_0_.

The evolution of the phase-field variables is described by

(6) τϵ∂ϕ∂t=ϵ(∇·∂a(ϕ,∇ϕ)∂∇ϕ−∂a(ϕ,∇ϕ)∂ϕ)−1ϵ∂w(ϕ)∂ϕ +∂s(e,ϕ)∂ϕ.

The thermodynamic relation *e* = *f* + *Ts* gives us ∂*s*(*e*, *ϕ*)/∂*ϕ* = −(1/*T*)(∂*f*(*T*, *ϕ*)/∂*ϕ*), detailed in [24]. Here is the bulk free energy density

(7) f(T,ϕ)=∑α∈{s,ℓ}Lα(T)T−TmTmh(ϕα) +∑α∈{s,ℓ}(∫TmTsvα(T~)dT~−T∫TmTsvα(T~)dT~T~)h(ϕα),

where *h*(*ϕ*) is a monotonous function on [0,1] with *h*(0) = 0, *h*(1) = 1 and *h*′(0) = *h*′(1) = 0. We chose *h*(*ϕ*) = *ϕ* ^3^(6*ϕ* ^2^ − 15*ϕ* + 10). *L* ^*α*^(*T*) is the latent heat and $s_{v}^{\alpha }(T)=\rho^{\alpha }(T){\tilde{c}}_{v}^{\alpha }(T)$ is the volumetric heat capacity both depending on the temperature *T*. *T*_*m*_ is the melting temperature.

For the isothermal case we can define two constants *f*(*T*, *ϕ*) = :∑_*α*∈{s,*ℓ*}_ *C*_*T*_^*α*^ *h*(*ϕ*_*α*_) and consider here that the free energy contribution to the equation of motion (6) is a constant term ∂*f*/∂*ϕ* = (*C*_*T*_^s^ − *C*_*T*_^*ℓ*^)*h*′(*ϕ*); note that *h*′(*ϕ*_s_) = −*h*′(*ϕ*_*ℓ*_).

Equation (6) is solved by a time dependent forward euler scheme with a second order spatial discretisation on a regular Cartesian grid.

## 4. Initial Configuration and Calibration

### 4.1. Preparation of the Sample

Growth simulations begin from a configuration made of a stabilized crystalline spherical cluster embedded in the melt. The preparation of the initial configuration involves a sequence of NpT simulation runs. First the crystalline solid phase is equilibrated at *p* = 0 starting from atoms arranged in a perfect fcc structure in a cubic box with an initial density close to the experimental value at room temperature (*ρ* = 8.9 g/cm^3^). The temperature in this step is chosen equal to the one used later in the simulation of growth. In the second step two regions are defined in the simulation box: the melting zone and the atoms in the crystalline phase (which is chosen here as a sphere of radius *r* = 25 Å). The region outside the crystalline zone is melted by increasing the temperature well above the melting point while the atoms in the solid region are constrained to remain at fixed positions. The temperature is linearly increased while an isotropic barostat is applied. In the next step, the stable melt at high temperature is cooled down to the temperature of the crystal. The melt reaches a metastable (undercooled) state. At the end of this run, a crystalline cluster embedded in the liquid phase at the same temperature is obtained. In the last step, the relaxation of the atoms at the interface is made by an NpT simulation of some picoseconds in which all particles are allowed to move.

### 4.2. Local Order Parameter

At microscopic level a configuration is described by positions and velocities of particles at a given time. The crystalline and liquid regions in this configuration can be identified by a suitable definition of the local order parameter, a function of the coordinates of a particle and its neighbors which adopts different values in the crystalline and in the disordered liquid phases. Here we use the bond local order parameter *q*_6_ *q*_6_ [25, 26]

(8) $$\begin{array}{c} q_{6}q_{6}\left(i\right):=\frac{1}{Z_{i}}\overset{Z_{i}}{\underset{j=1}{\sum }}q_{6}\left(i\right)\cdot q_{6}\left(j\right). \end{array}$$

The inner product in this sum is given by

(9) $$\begin{array}{c} q_{6}\left(i\right)\cdot q_{6}\left(j\right)=\overset{6}{\underset{m=-6}{\sum }}{\tilde{q}}_{6m}\left(i\right){\tilde{q}}_{6m}{\left(j\right)}^{*} \end{array}$$

with

(10) $$\begin{array}{c} {\tilde{q}}_{6m}\left(i\right)={\overset{-}{Q}}_{6m}\left(i\right){\left(\overset{6}{\underset{m=-6}{\sum }}{\left|{\overset{-}{Q}}_{6m}\left(i\right)\right|}^{2}\right)}^{-1/2}, \end{array}$$

where ${\overset{-}{Q}}_{6m}(i)=(1/Z_{i})\sum_{j=1}^{Z_{i}}Y_{6m}(\theta ({\vec{r}}_{ij}),\phi ({\vec{r}}_{ij}))$, *Z*_*i*_ is the number of neighboring atoms within a cut-off radius of 3.36 Å, and *Y*_6*m*_ are 6th order spherical harmonic functions.

In order to obtain an equivalent initial configuration for the PF method, the local order parameter values given at atom positions are mapped on a regular grid with cell-width of 1 Å. The order parameter in a given grid point is given by the average of all particles contained in cell **u** = (*i*, *j*, *k*) and its first two neighbor cells **v** = (*m*, *n*, *l*) with |**u** − **v** | <2.

The order parameter values obtained from MD simulation were scaled so that the volume of the crystal cluster is equal in the coarse-grained and in the original initial configurations. The distribution of the order parameter in the bulk phases is Gaussian centered at *μ* with standard deviation *σ*. The definition of order parameter in the phase-field method is as follows: *ϕ* = 0 if *q*_6_ *q*_6_ < *a* : = *μ*_*ℓ*_ − 2*σ*_*ℓ*_, and *ϕ* = 1 if *q*_6_ *q*_6_ > *b* : = *μ*_s_ + 2*σ*_s_ and *ϕ* = (*q*_6_ *q*_6_ − *a*)/(*b* − *a*) otherwise.

### 4.3. Planar Growth

An analytic formulation of the phase-field method in the sharp interface limit assumes a temperature gradient at the interface. The formulation reads

(11) $$\begin{array}{c} \Delta T=\beta v+\kappa \Gamma ; \end{array}$$

here Δ*T* denotes the undercooling in the interface, *v* is the velocity, and *β* is a kinetic coefficient. The curvature *κ* of a two dimensional interface is the sum of the two principal curvatures 1/*r*_1_,  1/*r*_2_; so *κ* = 2/*r* if *r*_1_ = *r*_2_ = *r*. The Gibbs-Thomson-coefficient Γ = *σT*/*L* with *σ* surface tension and *L* being the latent heat.

In equilibrium, that is, for *v* = 0, (11) reads as Δ*T* = *κ*Γ = Γ/*r* (in 2D). It follows that the critical radius *r*_*c*_ = Γ/Δ*T*. A small spherical cluster with radius *r*_*c*_ is in equilibrium. For example, at *T* = 1550 K the critical radius *r*_*c*_ = 9.7 Å. A cluster smaller than *r*_*c*_ melts. The phase-field simulations corroborate this result: for *T* = 1550 K the nucleus grows for *r* = 10 Å and melts for *r* = 9.5 Å.

For a planar interface (11) is simplified to Δ*T* = *βv*, taking account of the absence of curvature. The kinetic coefficient adapted from MD is *k* = 1/*β*. So the phase-field relaxation parameter is

(12) $$\begin{array}{c} \tau \left(\hat{n}\right)=\frac{L\left(T\right)}{TT_{m}k\left(\hat{n}\right)}=\frac{\tau_{0}}{k\left(\hat{n}\right)}. \end{array}$$

**Figure 1:**
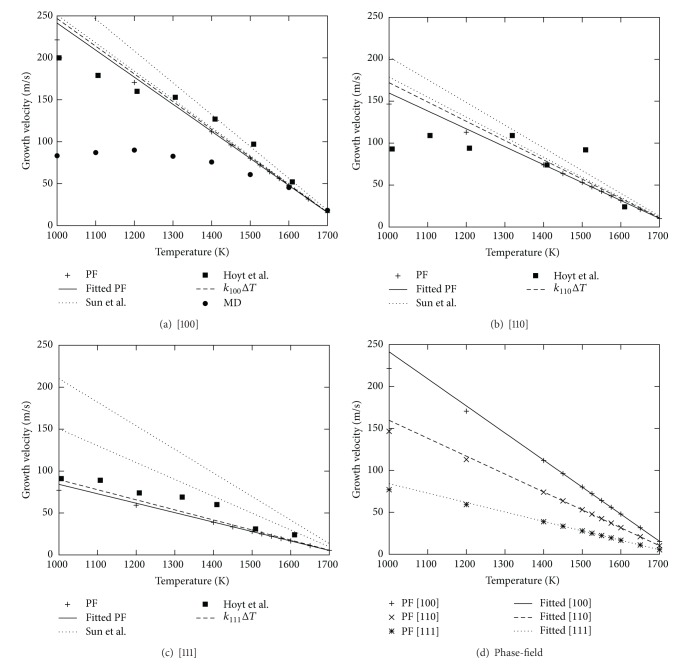
Growth velocity of a planar front at different temperatures for the interface orientations (a) [100], (b) [110], and (c) [111] in comparison to molecular dynamic results. The values of the kinetic coefficient can be found in Table 1. (d) shows the phase-field results for all orientations.

Simulations of a planar front in comparison to the analytic solution and molecular dynamics simulations are shown in Figure 1. Additionally we list the kinetic coefficient in Table 1. Both methods converge at low undercooling to the solution given by (11). At higher undercooling velocities obtained from PF simulations still exhibit a linear dependence with undercooling Δ*T*; this is given in the range from 1400 K to *T*_*m*_; the fit of the kinetic coefficient is made in this range and agrees with the expected kinetics from (1).

The MD simulations indicate that this linearity is not more valid; the velocity is lower than that expected from extrapolation of the linear relation *v*_*I*_ = *k*Δ*T*. This effect can also be recognized in the results of Hoyt et al. [15]. Many effects influence the growth velocity of MD at higher undercooling, with the increase of order in the liquid bulk being the most important which leads to the formation of small crystalline clusters in the bulk liquid. The phase-field shows this nonlinearity only for very high undercoolings Δ*T* > 500 K.

## 5. Spherical Cluster Growth

**Figure 2:**
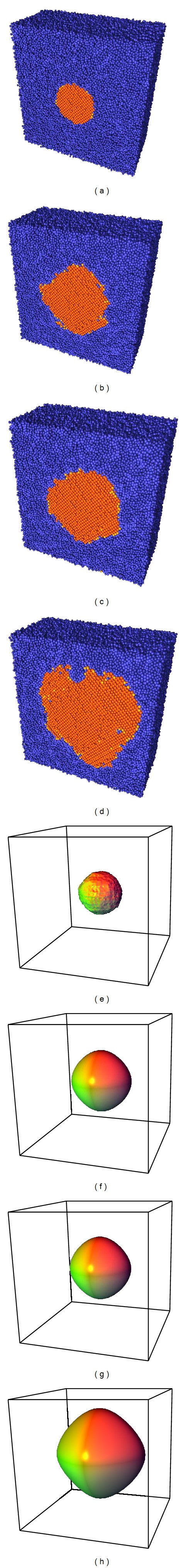
Snapshots of the simulations at 1550 K. MD simulations at (a) initial time 80 ps, (c) 100 ps, and (d) 220 ps. The evolution of the crystalline cluster from PF simulations with the same volume is shown in the sequence from (e) to (h).

**Figure 3:**
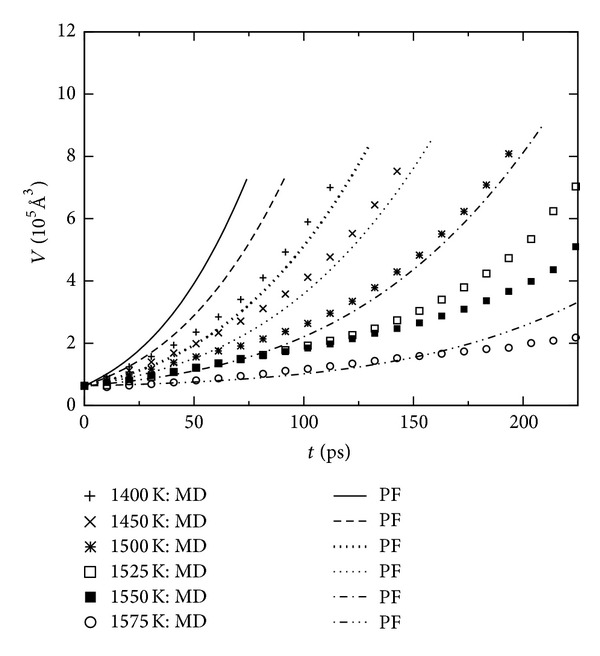
Evolution of the crystalline cluster volume at different temperatures. MD results are the average over 8 independent simulation runs.

**Figure 4:**
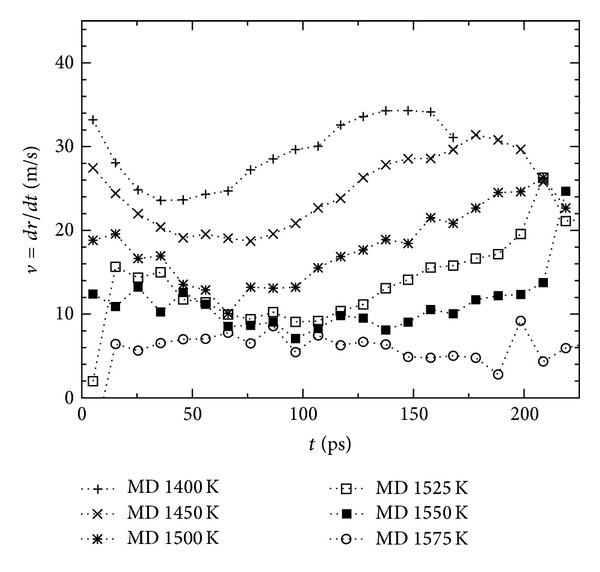
Evolution of the radial velocity at different temperatures obtained from MD simulations.

**Figure 5:**
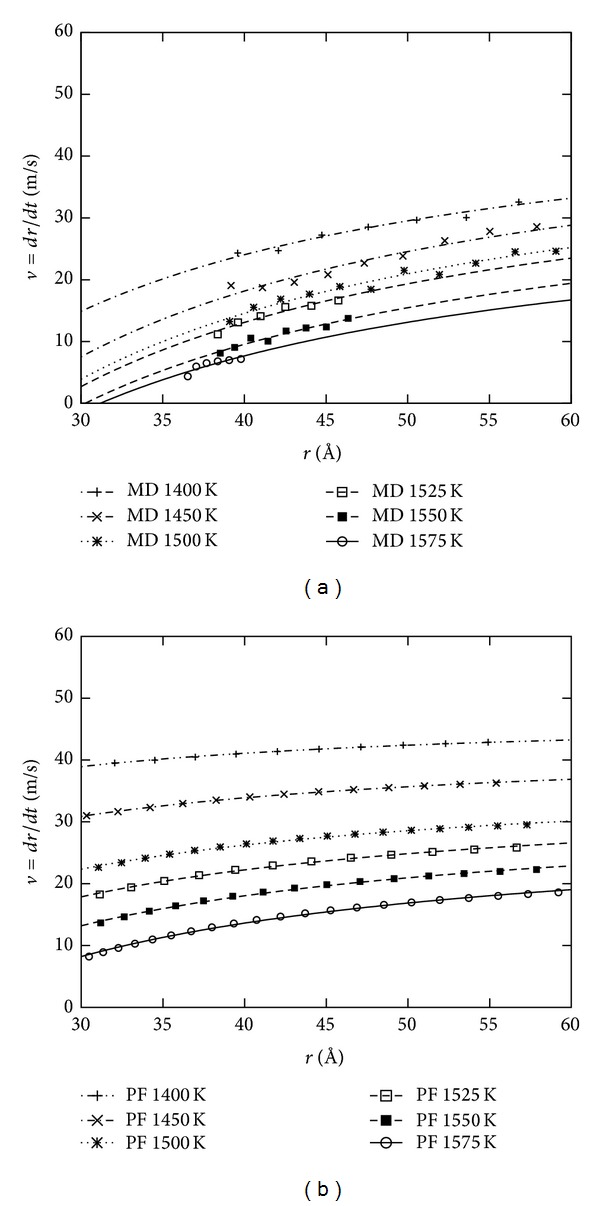
Evolution of the radial velocity at different temperatures. Molecular dynamic (a) and phase-field (b). In lines the fits *v*_*T*_(*r*): = *a*_*T*_ + *b*_*T*_/*r*.

**Figure 6:**
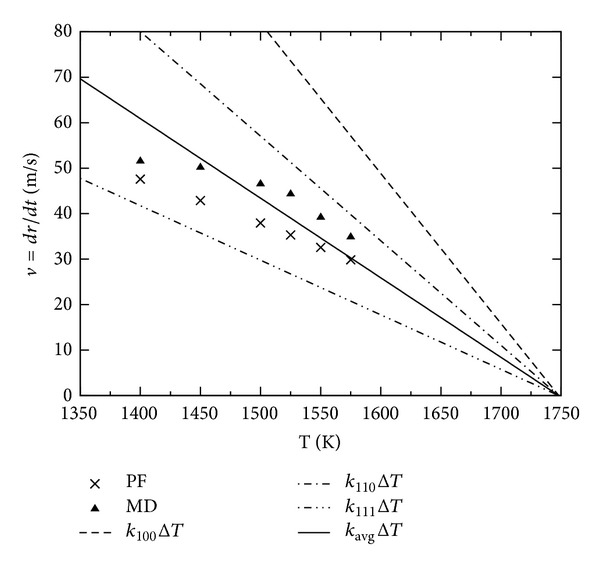
Asymptotic radial velocity versus temperature. Values of *v*_*∞*_ are obtained from the asymptotic limit of the fits shown in Figure 5.

Growth simulations starting from the samples prepared as described in Section 4.1 are performed using both MD and PF methods. At microscopic level NpT simulations are performed at *p* = 0 using the Andersen thermostat and barostat. The number of particles is *N* = 256000 which corresponds to a cubic box of length of about 145 Å. The initial radius of the spherical crystalline cluster is 25 Å which corresponds to about 5.5 · 10^3^ particles (see Figure 2(a)); that is much larger than the size of the critical cluster at *T* = 1500 K which is made of about 10^3^ particles [27]. For *T* ≥ 1600 K the initial size of the cluster is lower than the critical value and it melts as expected. PF simulations are performed imposing the isothermal condition starting with the same configuration as in MD but converted as described in Section 4.2. Results for the evolution of the cluster volume are shown in Figure 3; snapshots from the MD and PF simulations are shown in Figure 2. At moderate undercooling (*T* > 1550 K) the MD and PF show comparable growth rates. For higher undercooling the growth in the phase-field model is notably faster than in the MD method. The discrepancy becomes more pronounced at lower temperature and higher simulation time. Assuming that the cluster conserves its spherical shape during the first stage of growth we define the radius as *r* = (3*V*/4*π*)^1/3^ and the radial velocity as *v* = *dr*/*dt*.  Figure 4 shows the evolution of radial velocity in the MD simulation; three regimes can be identified; initially the velocity tends to decrease, then exhibits a linear increase, and finally decreases again. The first decrease is due to the preparation method of the sample; the system relaxes while the interface is formed. Growth and relaxation cannot be decoupled; both occur simultaneously in this initial regime. In the second regime the growth velocity increases linearly in time. The decrease of velocity in the last regime is due to the finite size of the sample; when the cluster is large enough it interacts with its periodic images through the periodic boundaries of the simulation box and loses its spherical shape. In the PF simulation no extra relaxation regime is observed; the dynamic is determined by the initial definition after the interface is relaxated. Moreover, in the PF the cluster is not affected by finite size effects as in the MD simulation since no periodic boundaries are required. In comparison the PF exhibits an apparent linear increase from the beginning. This means that the starting radius of the nucleus is not the same for both simulation methods, so a comparison for the same radius instead of the time is more adequate.  Figure 5 shows the radial velocity versus cluster radius at different temperatures. Results obtained from PF simulations are well described by Gibbs-Thomson equation (11). The analytical velocity is *v* = *k*_avg_(Δ*T* − 2Γ/*r*). So we use *V*_*T*_(*r*) = *a*_*T*_ + *b*_*T*_/*r* to fit the data with the parameters *a*_*T*_ and *b*_*T*_ for each temperature *T*. We applied the fit in the region starting with *r* = 30 Å, where the PF has relaxated the interface. These fits are also applied for the MD results in the second regime of growth, that is, for crystalline clusters of size of about 40 Å≾*r*≾60 Å (the points in Figure 5). According to the Gibbs-Thomson equation the radial velocity reaches an asymptotic value (*v*_*T*,*∞*_ = *v*_*T*,*r*→*∞*_), an ideal situation where the cluster is large enough and conserves its shape. This limit cannot be directly obtained from simulations first because the simulations are performed in a finite volume and also because the crystalline cluster would adopt a nonspherical shape due to the anisotropy of the interfacial energy and growth coefficient. We estimate this hypothetical limit as a basis of comparison between the PF and MD simulations. For this purpose we extrapolate the fit and obtain *v*_*T*,*∞*_ = *a*_*T*_; one value is obtained at each temperature. The asymptotic radial velocities obtained from the PF and MD simulations are shown in Figure 6. The results of both methods lie between the linear relations *v*_100_ = *k*_100_Δ*T* and *v*_111_ = *k*_111_Δ*T*. For the MD the results show a similar behavior as observed in the planar growth case; the deviation from the linear relation at high undercooling observed in the PF simulations is due to the correction introduced in the kinetic coefficient. As shown in Section 4.3 the crystal grows in different crystal orientations with a different velocity. In the PF the kinetic anisotropy is responsible for this. The planar front simulations from Figure 1 show that the kinetics are very well reproducible from the PF simulations. If we assume that we have a perfect sphere the mean kinetic can be calculated as a surface area integral of the unit sphere over the kinetic coefficient; we get $k_{\text{avg}}=\int_{S^{2}}k(\hat{n})dA/\int_{S^{2}}1dA\approx 0.175$. So *v*(Δ*T*) = *k*_avg_Δ*T* is the excepted growth velocity for a sphere; it is shown as the solid line in Figure 6.

At this early stage of growing the crystal is very similar to a sphere. The preferred growing in [100] directions and the surface anisotropy formed the crystal to its equilibrium shape; for Ni this is similar to an octahedron; in the limit this shape has only [111] directions. So the area of the [100] orientations shrinks and the area of the [111] orientations increases; it follows that the growth rate is between *k*_avg_ and *k*_111_, specially below *k*_avg_.

We use the *sphericitys* as the measure for the sphericality of the crystal as follow. For a given volume *V* and a given surface area *S* of the crystal we calculate the radius *r* ^3^ = 3*V*/4*π* from the volume assuming a sphere and calculate the surface area *S*_*V*_ = 4*πr* ^2^ for a sphere. Then *s* : = *S*_*V*_/*S* [28].

The volume of the crystal is defined as *V* : = ∫_*Ω*_ *ϕdV* and the surface area as *S* : = ∫_*Ω*_||∇*ϕ*||*dV*; this surface area measurement method assumes a smooth nondeformed interface; this is not given for PF interfaces if there are driving forces like curvatures and undercoolings. We use a factor estimated for a sphere of radius 25 Å to correct this. The surface area is multiplied by this factor 1.089.

**Figure 7:**
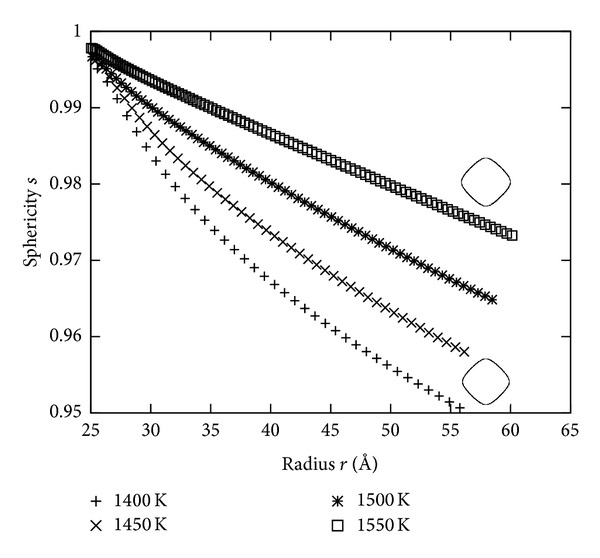
Sphericity of the crystal for different undercoolings. The contour shows the shape of the [100] cutting plane through the center of the crystal for *T* = 1400 K and 1550 K.

Figure 7 shows the sphericity over the radius; the crystal becomes non spherical for longer growing and faster for higher undercoolings. This means that the velocity of growing is below *k*_avg_ especially for higher undercoolings. The nonspherical shape is shown in Figure 2(h).

## 6. Conclusion

Many have shown the linking between MD and PF [16, 17]. So it is a common way to obtain MD parameters for PF simulations. The PF simulations on the natural PF scale, for example, mesoscopic scale, produce correct results compared with experiments [29, 30]. We have confirmed that a PF with MD parameters is correct even on the atomistic scale. This is not surprising, since we are very close to the sharp interface limits. The mesoscopic view at the atomistic scale provides the same growth rates as an atomic simulation method, although the atoms are not resolved in the PF. The fluctuations of the interface on the atomistic length scale will be investigated in a consecutive work.

However, you will not simulate too high undercooled melts, where the mesoscopic view of the PF will not consider all the nonlinearity from the MD interface. We have used the sphericity to measure the shape and explain the discrepancy to the analytic linear velocity.

While it is known that PF works correctly on mesoscopic scale and with this study it is shown that PF also works on the atomistic scale, one is able to do multiscale simulations starting on the atomistic scale with one simulation method.

## Figures and Tables

**Table 1:** Kinetic coefficient of Ni in m/sK from molecular-dynamics simulations and phase-field simulation.

Interface	From (1)	PF	Sun et al. [12]
[100]	0.33	0.32	0.358 ± 0.022
[110]	0.23	0.21	0.255 ± 0.016
[111]	0.12	0.11	0.241 ± 0.04

## References

[1] Chen L-Q (2002). Phase-field models for microstructure evolution. *Annual Review of Materials Science*.

[2] Boettinger WJ, Warren JA, Beckermann C, Karma A (2002). Phase-field simulation of solidification. *Annual Review of Materials Science*.

[3] Karma A, Rappel W-J (1998). Quantitative phase-field modeling of dendritic growth in two and three dimensions. *Physical Review E*.

[4] Nestler B, Garcke H, Stinner B (2005). Multicomponent alloy solidification: phase-field modeling and simulations. *Physical Review E*.

[5] Gránásy L, Pusztai T, Warren JA (2004). Modelling polycrystalline solidification using phase field theory. *Journal of Physics: Condensed Matter*.

[6] Karma A (1994). Phase-field model of eutectic growth. *Physical Review E*.

[7] Nestler B, Wheeler AA (2000). A multi-phase-field model of eutectic and peritectic alloys: numerical simulation of growth structures. *Physica D*.

[8] Nestler B, Wheeler AA, Ratke L, Stöcker C (2000). Phase-field model for solidification of a monotectic alloy with convection. *Physica D*.

[9] Zhu MF, Hong CP (2001). A modified cellular automaton model for the simulation of dendritic growth in solidification of alloys. *ISIJ International*.

[10] Zhu P, Smith RW (1992). Dynamic simulation of crystal growth by Monte Carlo method-I. Model description and kinetics. *Acta Metallurgica et Materialia*.

[11] Plapp M, Karma A (2000). Multiscale finite-difference-diffusion Monte-Carlo method for simulating dendritic solidification. *Journal of Computational Physics*.

[12] Sun DY, Asta M, Hoyt JJ (2004). Kinetic coefficient of Ni solid-liquid interfaces from molecular-dynamics simulations. *Physical Review B*.

[13] Kerrache A, Horbach J, Binder K (2008). Molecular-dynamics computer simulation of crystal growth and melting in Al_50_Ni_50_. *EPL*.

[14] Rozas RE, Horbach J (2011). Capillary wave analysis of rough solid-liquid interfaces in nickel. *EPL*.

[15] Hoyt JJ, Asta M, Karma A (2003). Atomistic and continuum modeling of dendritic solidification. *Materials Science and Engineering R*.

[16] Bragard J, Karma A, Lee YH, Plapp M (2002). Linking phase-field and atomistic simulations to model dendritic solidification in highly undercooled melts. *Interface Science*.

[17] Nestler B, Selzer M, Danilov D (2009). Phase-field simulations of nuclei and early stage solidification microstructures. *Journal of Physics: Condensed Matter*.

[18] Tegze G, Pusztai T, Tóth G (2006). Multiscale approach to CO_2_ hydrate formation in aqueous solution: phase field theory and molecular dynamics. Nucleation and growth. *The Journal of Chemical Physics*.

[19] Foiles SM (1985). Application of the embedded-atom method to liquid transition metals. *Physical Review B*.

[20] Rozas RE, Horbach J A comparison of EAM models of Ni close to the melting temperature.

[21] Zinov’yev VY, Polev VF, Taluts SG, Zinov’yeva GP, Il’inykh SA (1986). Diffusivity and thermal conductivity of 3D-transition metals in solid and liquid states. *Physics of Metals and Metallography*.

[22] Nagata K, Fukuyama H, Taguchi K, Ishii H, Hayashi M (2003). Thermal conductivity of molten Al, Si and Ni measured under microgravity. *High Temperature Materials and Processes*.

[23] Hoyt JJ, Asta M, Haxhimali T (2004). Crystal-melt interfaces and solidification morphologies in metals and alloys. *MRS Bulletin*.

[24] Garcke H, Nestler B, Stinner B (2004). A diffuse interface model for alloys with multiple components and phases. *SIAM Journal on Applied Mathematics*.

[25] Steinhardt PJ, Nelson DR, Ronchetti M (1983). Bond-orientational order in liquids and glasses. *Physical Review B*.

[26] ten Wolde PR, Ruiz-Montero MJ, Frenkel D (1995). Numerical evidence for bcc ordering at the surface of a critical fcc nucleus. *Physical Review Letters*.

[27] Bokeloh J, Rozas RE, Horbach J, Wilde G (2011). Nucleation barriers for the liquid-to-crystal transition in Ni: experiment and simulation. *Physical Review Letters*.

[28] Wadell H (1935). Volume, shape, and roundness of quartz particles. *The Journal of Geology*.

[29] Echebarria B, Folch R, Karma A, Plapp M (2004). Quantitative phase-field model of alloy solidification. *Physical Review E*.

[30] Gurevich S, Karma A, Plapp M, Trivedi R (2010). Phase-field study of three-dimensional steady-state growth shapes in directional solidification. *Physical Review E*.

